# Metabolic reprogramming in intervertebral disc degeneration: mechanisms and therapeutic opportunities

**DOI:** 10.3389/fcell.2026.1811737

**Published:** 2026-04-13

**Authors:** Qing Lu, Caiyou He

**Affiliations:** 1 Department of Emergency, Honghui Hospital, Xi’an Jiaotong University, Xi’an, Shaanxi, China; 2 Health Science Center, Xi’an Jiaotong University, Xi’an, Shaanxi, China

**Keywords:** extracellular matrix degradation, intervertebral disc degeneration, metabolic reprogramming, mitochondrial dysfunction, therapeutic targets

## Abstract

Intervertebral disc degeneration (IVDD) is a major cause of chronic low back pain and disability and imposes a substantial socioeconomic burden. Increasing evidence indicates that metabolic reprogramming is closely involved in the initiation and progression of IVDD. In this review, we summarize the major pathological processes associated with IVDD, including apoptosis, autophagy dysregulation, oxidative stress, inflammatory responses, and extracellular matrix (ECM) degradation. We further discuss alterations in glucose, lipid, and amino acid metabolism, with particular emphasis on the contribution of mitochondrial dysfunction to metabolic imbalance in disc cells. In addition, we outline the genetic, environmental, and signaling factors that regulate metabolic reprogramming, including pathways such as mTOR and AMPK. Finally, we review emerging metabolism-related therapeutic strategies, including metabolic enzyme modulation, antioxidants, and mitochondrial protectors. Collectively, current evidence suggests that metabolic reprogramming is an important component of IVDD pathogenesis and may provide a useful framework for the development of targeted therapeutic approaches.

## 1 Introduction

Intervertebral disc degeneration (IVDD) is a major contributor to chronic low back pain and lower limb symptoms, including pain, numbness, and impaired mobility, and it markedly affects quality of life and work capacity (Liu X. W. et al., 2023). As a common age-related musculoskeletal disorder, IVDD is increasingly recognized as an important public health problem worldwide (Risbud and Shapiro, 2014). The burden of low back pain associated with disc degeneration is substantial, with major healthcare expenditures and productivity loss reported globally (Fatoye et al., 2023). These clinical and socioeconomic consequences underscore the need for a better understanding of IVDD pathogenesis and for more effective preventive and therapeutic strategies.

The pathophysiology of IVDD is complex and multifactorial. Previous studies have shown that disc degeneration is associated with apoptosis, autophagy imbalance, chronic inflammation, oxidative stress, mitochondrial dysfunction, and extracellular matrix (ECM) degradation (Chen X. et al., 2023). These processes interact with each other and collectively disrupt disc cell homeostasis, tissue structure, and biomechanical function. Traditional studies have largely focused on mechanical stress, inflammatory signaling, and matrix catabolism, which have greatly advanced our understanding of disc degeneration (Bhujel et al., 2022). However, these mechanisms alone do not fully explain the heterogeneity of IVDD progression or the coordinated molecular changes observed during degeneration.

In recent years, increasing attention has been directed toward the role of cellular metabolism in degenerative diseases. Metabolic reprogramming refers to the adaptive remodeling of metabolic pathways in response to environmental stress, pathological stimuli, or altered cellular demands (Pavlova and Thompson, 2016). Although this concept was initially established in cancer biology, it is now recognized as broadly relevant to multiple pathological conditions, including inflammatory, cardiovascular, neurodegenerative, and age-related disorders (DeBerardinis and Thompson, 2012). In the context of IVDD, the intervertebral disc exists in a unique microenvironment characterized by hypoxia, limited nutrient supply, acidic pH, and restricted vascularization. These features make disc cells particularly dependent on metabolic adaptation for survival and functional maintenance.

Emerging evidence suggests that metabolic reprogramming is closely involved in IVDD and affects several interconnected pathways, including glucose metabolism, lipid metabolism, amino acid metabolism, and mitochondrial bioenergetics. For example, hypoxia-associated metabolic adaptation influences glycolytic activity in nucleus pulposus (NP) cells, while oxidative stress and mitochondrial impairment further aggravate metabolic imbalance and cell dysfunction s (Wang Z. et al., 2023). In addition, recent studies have highlighted the relevance of immunometabolism and metabolomic alterations in degenerated disc tissues, further supporting the concept that metabolic dysregulation is not merely a secondary consequence of degeneration but may actively contribute to disease progression (Schoors et al., 2015). These observations indicate that metabolism-centered mechanisms may provide a useful integrative framework for understanding how inflammation, oxidative stress, cell death, and matrix degradation are linked during IVDD.

Based on these considerations, this review summarizes the current understanding of metabolic reprogramming in IVDD. We first outline the major pathological mechanisms involved in disc degeneration and then discuss the metabolic alterations associated with glucose, lipid, and amino acid pathways, as well as mitochondrial dysfunction. We further examine the regulatory factors and signaling pathways that shape metabolic remodeling and highlight potential therapeutic opportunities targeting these processes. By integrating recent evidence, this review aims to provide a clearer overview of the metabolic basis of IVDD and to support future investigation of metabolism-targeted interventions.

## 2 Pathological mechanisms of IVDD

### 2.1 Normal structure and function of the intervertebral disc

A healthy intervertebral disc consists of the nucleus pulposus, annulus fibrosus (AF), and cartilage endplates, which work together to maintain spinal flexibility and stability. The nucleus pulposus is primarily composed of water-rich proteoglycans and collagen fibers, functioning to absorb and dissipate mechanical pressure (Sun et al., 2022). The AF consists of multiple layers of collagen fibers that provide mechanical strength and flexibility, preventing nucleus pulposus herniation (Vergroesen et al., 2015). The cartilage endplates cover the upper and lower surfaces of the vertebral bodies, serving to connect the vertebrae and discs while supplying nutrients to the nucleus pulposus via diffusion (Raj, 2008). The normal function of these structures relies on the integrity of the ECM and the health of the cells. Recent studies have shown that maintaining the osmotic pressure and pH levels within the nucleus pulposus is critical for cellular homeostasis and ECM synthesis, and any deviations from these parameters may lead to disc degeneration (Francisco et al., 2022).

Advancements in high-resolution imaging technologies have allowed for a deeper understanding of the intricate fiber structure of the AF, which is crucial for distributing mechanical loads and preventing disc herniation (Molinos et al., 2015). The cartilage endplates not only act as a barrier and interface between the vertebral bodies and discs but also play a key role in nutrient transport. Given the avascular nature of the intervertebral disc, the diffusion of nutrients and the removal of waste occur through the cartilage endplates, which are essential for maintaining disc health (Feng et al., 2016). Additionally, the balance between ECM synthesis and degradation is regulated by matrix metalloproteinases (MMPs) and their inhibitors (TIMPs), which are crucial for maintaining the integrity of the disc. Disruption of this balance can accelerate the degenerative process.

### 2.2 Pathological changes in IVDD

**FIGURE 1:**
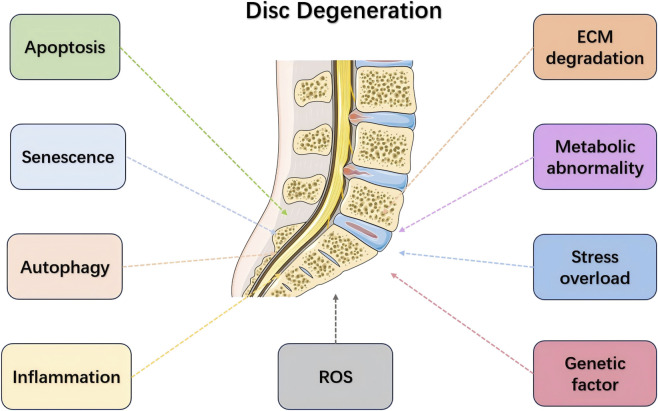
Pathologic mechanisms of intervertebral disc degeneration (IVDD). This figure summarizes the major pathological processes involved in IVDD, including apoptosis and autophagy dysregulation, inflammatory responses, extracellular matrix (ECM) breakdown, and oxidative stress, as well as their contributions to disc structural deterioration and cellular dysfunction.

The pathological changes in IVDD include apoptosis and autophagy, inflammatory responses, ECM degradation, and oxidative stress. These factors collectively lead to the loss of intervertebral disc structure and function (Figure 1).

#### 2.2.1 Apoptosis and autophagy

The increase in apoptosis and impairment of autophagic function in intervertebral disc cells are the primary pathological changes in IVDD. Studies have demonstrated the involvement of both intrinsic and extrinsic apoptotic pathways in IVDD. The extrinsic apoptotic pathway is activated when ligands such as FasL or TNF-α bind to apoptotic receptors like Fas or TNF receptor 1, leading to the activation of the death domain, which recruits the Fas-associated death domain (FADD). This further activates Caspase-8, initiating a caspase cascade that ultimately results in apoptosis. The intrinsic apoptotic pathway is closely related to mitochondrial dysfunction. Key regulatory proteins include members of the Bcl-2 family, particularly Bax and Bak, which increase the permeability of the mitochondrial outer membrane during apoptosis, leading to the release of cytochrome C into the cytosol. Cytochrome C binds with Apaf-1 to form the apoptosome, which activates Caspase-9, subsequently activating downstream effectors such as Caspase-3 (Wang et al., 2020). Additionally, research indicates that epigenetic mechanisms and oxidative stress also play roles in regulating apoptosis during disc degeneration (Kang et al., 2023).

Autophagy plays a dual role in maintaining cellular health, functioning as both a protective mechanism and, under certain conditions, a trigger for cell death. On one hand, autophagy helps NP and AF cells survive under adverse conditions such as hypoxia, nutrient deprivation, and mechanical stress, while clearing excessive reactive oxygen species (ROS) generated by mitochondrial damage, thereby reducing oxidative stress-induced DNA and protein damage. On the other hand, excessive or dysregulated autophagy can be interconnected with apoptosis, leading to autophagic cell death. For instance, under conditions of excessive autophagy, Beclin-1 competitively binds to Bcl-2 proteins, increasing the formation of autophagosomes and inducing cell death (Kritschil et al., 2022). Additionally, excessive autophagy can suppress Beclin-1 cleavage by Caspase-8, thereby activating apoptosis in NP cells through extrinsic apoptotic pathways (Zhang et al., 2020). The role of autophagy continues to be explored, with recent studies revealing that SUV39H2 promotes intervertebral disc degeneration by accelerating the process through lysine methylation of PPP1CA, which inhibits TFEB-mediated autophagy regulation (Liang et al., 2023). Moreover, excessive mechanical load activates PKN, leading to phosphorylation of KRT8, which inhibits autophagosome initiation, ultimately accelerating disc degeneration.

Beyond their direct effects on disc cell survival, dysregulated apoptosis and autophagy can further enhance inflammatory signaling, thereby contributing to the progression of IVDD.

#### 2.2.2 Inflammatory responses

In addition to the inflammatory mechanisms triggered by mechanical stress and extracellular matrix damage, recent studies have identified hypoxic environments as a source of inflammatory injury to NP cells, contributing to IVDD. Under hypoxic conditions, HIF1A (hypoxia-inducible factor 1-alpha) upregulates BNIP3 expression, promoting BNIP3-LC3 interaction to initiate mitophagy, clearing damaged mitochondria, reducing ROS accumulation, and suppressing apoptosis, thereby maintaining metabolic homeostasis and mitochondrial function in NP cells (He et al., 2021). Other research has also highlighted a protective mechanism of HIF1A in NP cells, demonstrating that mechanical compression induces HIF1A to upregulate LC3-II and Beclin-1, enhancing autophagic activity to clear damaged organelles and misfolded proteins, thereby inhibiting apoptosis pathways induced by compression (Madhu et al., 2023).

In parallel, immune cell infiltration plays a multifaceted role in disc degeneration. For instance, rupture of the AF allows macrophages to infiltrate and polarize into either pro-inflammatory or anti-inflammatory phenotypes, depending on the microenvironment. M1 macrophage-derived exosomes, through the LCN2/NF-κB signaling axis, exacerbate NP cell senescence and inflammation, thereby accelerating disc degeneration (Fan et al., 2024). In contrast, CD206 (+) M2 macrophages provide partial protection by targeting R-spondin-2, promoting anti-inflammatory responses and tissue repair, thereby mitigating disc degeneration (Li et al., 2024). Additionally, immune cells can promote the release of substance P and norepinephrine, inducing the ingrowth of sensory and sympathetic nerve fibers into the NP and AF, which contributes to chronic low back pain or sciatica (Sun et al., 2022). Furthermore, pyroptosis, induced by NLRP3 inflammasomes, has been shown to play a regulatory role in disc degeneration. This process involves NLRP3 activation of caspase-1, leading to the cleavage of Gasdermin D, which results in NP cell membrane perforation and the release of pro-inflammatory factors (Jiang et al., 2018).

Persistent inflammation not only disrupts the disc microenvironment but also accelerates extracellular matrix catabolism, ultimately promoting structural deterioration of the intervertebral disc.

#### 2.2.3 ECM degradation

The accelerated degradation of collagen and proteoglycans, the main components of the ECM, is one of the key factors contributing to the structural and functional loss of the intervertebral disc. During IVDD, the expression of MMPs and ADAMTS proteases increases, leading to the accumulation of ECM degradation products, thereby compromising the integrity and functionality of the NP and AF. In addition to the involvement of the MMPs family and inflammatory cytokines (IL-1β and TNF-α) in activating oxidative stress and inflammation, recent studies have revealed that PIEZO1, a mechanosensitive ion channel, can activate the NF-κB pathway in response to mechanical force. This, in turn, induces the upregulation of the ECM protein Periostin, forming a self-amplifying NF-κB-Periostin feedback loop that accelerates NP cell senescence and disc degeneration (Wu et al., 2022). Furthermore, extracellular vesicles containing GLRX3, combined with a redox-balanced hydrogel, have been shown to modulate NP cell redox homeostasis and inhibit ROS-induced ECM degradation, thereby preventing NP cell senescence (Liu C. et al., 2023).

ECM degradation is also closely linked to autophagy and apoptosis. BNIP3, a key receptor for mitophagy, has been found to maintain mitochondrial function and metabolic homeostasis in NP cells by promoting the clearance of damaged mitochondria, reducing ROS accumulation and ECM degradation, thus alleviating disc degeneration (Madhu et al., 2023). Moreover, TAK-715 has been shown to inhibit the p38 MAPK signaling pathway, reducing IL-1β-induced NP cell apoptosis and ECM degradation by lowering MMPs expression, thereby slowing disc degeneration both *in vitro* and *in vivo* (Wang K. et al., 2023).

In turn, progressive ECM breakdown further impairs disc cell homeostasis and may exacerbate oxidative stress, forming a vicious cycle that accelerates disc degeneration.

#### 2.2.4 Oxidative stress

In addition to apoptosis, inflammation, and ECM degradation, oxidative stress is another major contributor to IVDD progression. Oxidative stress is a key pathological mechanism in the progression of IVDD. The generation of ROS and the activation of inflammation can lead to mitochondrial-induced apoptosis, pyroptosis, cellular senescence, and dysregulated autophagy, all of which accelerate ECM degradation. In IVDD patients, the levels of ROS in NP and AF cells are significantly elevated. Research has shown that ROS levels in NP cells from IVDD patients are nearly 50% higher than those in healthy controls (Zhao et al., 2020). Antioxidant therapies have demonstrated potential in alleviating IVDD symptoms. Recent studies have discovered that the deubiquitinating enzyme USP11 can stabilize Sirt3 through deubiquitination, reducing iron accumulation and lipid peroxidation. This regulation of oxidative stress-induced ferroptosis provides a protective effect against disc degeneration (Zhu et al., 2023a).

## 3 Basic concepts of metabolic reprogramming

### 3.1 Definition of metabolic reprogramming

Metabolic reprogramming refers to the process by which cells adjust their metabolic pathways and energy utilization in response to specific environmental or pathological conditions, allowing them to adapt to new physiological demands or external pressures. This phenomenon is widespread in various physiological and pathological states, such as cancer, inflammation, tissue regeneration, and aging (Pavlova and Thompson, 2016). In recent years, significant progress has been made in understanding metabolic reprogramming, particularly in cancer, stem cell biology, immunology, tissue repair, and other fields. Under disease conditions, cells often reprogram their metabolism to adapt to pathological environments and ensure survival. For example, cancer cells undergo metabolic reprogramming to support rapid proliferation and survival; under hypoxic conditions, cells shift metabolic pathways to adapt to oxygen deprivation (Vander Heiden et al., 2009).

In tumors, metabolic reprogramming is a hallmark feature. Even in the presence of sufficient oxygen, tumor cells preferentially generate energy through glycolysis rather than oxidative phosphorylation, a phenomenon known as the Warburg effect. This metabolic shift allows for rapid ATP production, providing ample energy for tumor cell growth and division. Additionally, cancer cells alter glutamine metabolism to supply energy and produce intermediates necessary for biosynthesis. Targeted therapy that inhibits glutamine metabolism has been shown to significantly reduce the supply of amino acids, particularly aspartate, thereby impairing protein synthesis and arresting cancer cell growth (DeBerardinis and Chandel, 2016). Recent studies also revealed that inhibiting fatty acid synthesis by targeting fatty acid synthase (FASN), which cancer cells heavily rely on, can weaken the integrity of cell membranes and disrupt signal transduction, ultimately reducing the proliferative capacity of cancer cells and inducing apoptosis (Cheng et al., 2018).

In inflammatory diseases, metabolic reprogramming has been observed to sustain the energy needs of immune cells and promote the production of pro-inflammatory mediators. For instance, classically activated M1-polarized macrophages increase glycolysis and the pentose phosphate pathway to support the production of ROS and cytokines. In contrast, alternatively activated M2-polarized macrophages rely on oxidative phosphorylation and fatty acid oxidation, highlighting the multifaceted role of metabolic reprogramming in immune responses (Shen et al., 2022).

In degenerative joint diseases such as osteoarthritis, metabolic reprogramming is believed to be a key mechanism in altering chondrocyte energy metabolism. Chondrocytes, due to the hypoxic environment within joints, may shift from oxidative phosphorylation to glycolysis, leading to increased lactate production and joint cavity acidification. This metabolic shift may promote inflammation and cartilage degradation. Additionally, in osteoporosis, metabolic changes in osteoblasts and osteoclasts affect the dynamic balance between bone formation and resorption. Osteoblasts may exhibit decreased glycolytic activity, impairing their ability to form bone matrix, while osteoclasts may increase oxidative phosphorylation to enhance bone resorption (Kode et al., 2012).

These findings underscore the diversity of metabolic reprogramming in various diseases and highlight its potential as a therapeutic target. Metabolic reprogramming not only impacts energy metabolism but also involves adjustments in amino acid, lipid, and nucleotide metabolic pathways.

### 3.2 Major pathways of metabolic reprogramming

#### 3.2.1 Glycolysis

In various diseases, including IVDD, activation of the glycolytic pathway is a common metabolic alteration. Under normoxic conditions, cells primarily generate ATP through oxidative phosphorylation, whereas under hypoxia, they rely on glycolysis for energy production. However, studies have shown that certain pathological cells tend to favor glycolysis even in the presence of oxygen, a phenomenon known as “aerobic glycolysis” or the “Warburg effect”. In numerous studies, the glycolytic pathway has been implicated in the progression of IVDD. In the early stages of disc degeneration, cells enhance glycolytic activity through metabolic reprogramming to adapt to oxygen and nutrient deficiency. This process is mediated by the activation of hypoxia-inducible factor 1-alpha (HIF-1α), which upregulates the expression of key glycolytic enzymes such as hexokinase (HK), phosphofructokinase (PFK), and lactate dehydrogenase (LDH) (Francisco et al., 2023). Moreover, as mitochondrial dysfunction progresses during disc degeneration, the efficiency of oxidative phosphorylation declines, forcing cells to rely more heavily on glycolysis for energy. This metabolic shift is also closely associated with increased apoptosis and autophagy (Liu et al., 2024).

#### 3.2.2 TCA cycle

The tricarboxylic acid (TCA) cycle is the primary pathway for cellular energy production, and alterations in this cycle during metabolic reprogramming play a crucial role in IVDD (Martínez-Reyes and Chandel, 2020). The activity of the TCA cycle may be influenced by various factors such as hypoxia, oxidative stress, and inflammatory responses. Studies have revealed that levels of TCA cycle intermediates, such as citrate and succinate, are significantly reduced in the disc cells of IVDD patients, suggesting suppressed TCA cycle activity. This suppression could impair ATP production in NP cells and compromise their survival capacity (Bourgonje et al., 2020).

#### 3.2.3 Lipid metabolism

The reprogramming of lipid metabolism significantly impacts cell membrane structure and signal transduction. In IVDD, alterations in lipid metabolism may affect membrane fluidity and stability, thereby influencing cell function and survival. Moreover, lipid metabolic reprogramming is closely associated with inflammatory responses, as certain lipid metabolites, such as arachidonic acid and its derivatives, play pivotal roles in the inflammatory process (Jin et al., 2023).

#### 3.2.4 Amino acid metabolism

Changes in amino acid metabolism are essential for maintaining cellular energy balance and antioxidant capacity. Research indicates that certain amino acids, such as glutamine, play a crucial role in metabolic reprogramming by providing energy, maintaining redox balance, and participating in the synthesis of nucleotides and proteins to support cell survival (Martinez-Outschoorn et al., 2017). In IVDD, the reprogramming of amino acid metabolism enables cells to adapt to hypoxic and nutrient-deprived environments while protecting them from oxidative stress through antioxidant mechanisms (Lynch and Adams, 2014).

## 4 Specific mechanisms of metabolic reprogramming in IVDD

### 4.1 Glycolysis and lactic acid metabolism

#### 4.1.1 Lactic acid accumulation and its impact on IVDD

The hypoxic environment of the intervertebral disc forces cells to rely predominantly on glycolysis for energy metabolism, resulting in substantial lactate production, as confirmed by clinical data. The accumulation of lactate acidifies the ECM, impairing cellular function (Zhu et al., 2023b). This lactate accumulation lowers local pH, enhances MMP activity, accelerates ECM degradation, inhibits cell proliferation, and induces apoptosis, collectively exacerbating disc degeneration (Vo et al., 2013).

Recent studies further underscore lactate’s role in modulating immune responses within the disc microenvironment. Lactate functions as an immunosuppressant, dampening the activity of immune cells such as macrophages and T cells, thereby reshaping the local immune landscape (Zhao et al., 2024). This immunosuppressive effect may foster chronic inflammation, driving further tissue degeneration. Additionally, lactate influences the differentiation and function of disc cells, promoting a catabolic phenotype marked by increased ECM degradation and diminished anabolic activity (Wuertz et al., 2009).

#### 4.1.2 Related signaling pathways

Under hypoxic conditions, the reprogramming of glycolysis is driven by the activation of hypoxia-inducible factor HIF-1α. HIF-1α boosts cellular reliance on glycolysis by upregulating glucose transporters (GLUTs) and key glycolytic enzymes (Papandreou et al., 2006). In IVDD, prolonged activation of HIF-1α not only promotes lactate production but also regulates the expression of crucial genes, such as GLUT1, HK2, and LDHA, thereby influencing cellular activity and metabolic status (Zhu et al., 2023a). Furthermore, HIF-1α interacts with other signaling pathways, notably the NF-κB pathway, to modulate inflammatory responses within the disc. This interaction aggravates degeneration by increasing the release of pro-inflammatory cytokines—TNF-α, IL-1β, IL-6, and IL-17—and upregulating the expression of catabolic enzymes, including MMP-3, MMP-9, MMP-13, and ADAMTSs (Huang et al., 2022). Moreover, HIF-1α appears to facilitate angiogenesis within the disc by binding to VEGFR-2, which activates the PI3K/Akt and MAPK signaling pathways, thereby promoting endothelial cell proliferation and migration (Zhu et al., 2020).

### 4.2 Changes in theTCA cycle

#### 4.2.1 Role of the TCA cycle in IVDD

The TCA cycle is the central pathway for cellular energy production, and its disruption can lead to metabolic imbalances in disc cells. Research has shown that the levels of TCA cycle intermediates, such as citrate and succinate, are significantly reduced in the disc cells of IVDD patients, indicating impaired TCA cycle activity (Tendulkar et al., 2019). These alterations are closely linked to hypoxia and mitochondrial dysfunction, where insufficient ATP production and the accumulation of ROS trigger cellular senescence and apoptosis (Crean et al., 1997). Recent studies further suggest that the downregulation of TCA cycle activity in IVDD may stem from defective uptake and utilization of key substrates like pyruvate, which is essential for initiating the cycle (Schoepflin et al., 2017). Additionally, disruptions in the TCA cycle can lead to the accumulation of intermediates that activate pseudo-hypoxic signaling pathways, such as those mediated by HIF-1α, further driving a metabolic shift towards glycolysis (Mills and O'Neill, 2014).

#### 4.2.2 Regulation of key enzymes

Succinate dehydrogenase (SDH) and fumarate hydratase (FH) are key enzymes in the TCA cycle, and their expression and activity are altered in IVDD (Lynch and Adams, 2014). Studies have shown that the expression of SDH and FH is significantly decreased in the disc cells of IVDD patients, leading to metabolic disturbances of succinic acid and fumaric acid in the TCA cycle (Wang and Tontonoz, 2018). This reduction in enzyme activity not only affects ATP production but also leads to the accumulation of metabolic products, activating oxidative stress and inflammatory responses (Vander Heiden and DeBerardinis, 2017). The reduced activity of these enzymes not only compromises ATP production but also causes the accumulation of succinate and fumarate, which can act as oncometabolites. These accumulated metabolites have been shown to inhibit prolyl hydroxylase enzymes, stabilizing HIF-1α under normoxic conditions and contributing to an aberrant metabolic state characterized by increased oxidative stress and inflammation (Reid et al., 2017).

### 4.3 Reprogramming of lipid metabolism

#### 4.3.1 Role of lipid metabolism in IVDD

Lipid metabolism is essential for maintaining cell membrane integrity, signal transduction, and energy homeostasis. In healthy intervertebral discs, both glucose metabolism and lipid metabolism support the energy needs of NP and AF cells. Lipid metabolism encompasses two key processes: fatty acid oxidation (FAO), a major mitochondrial pathway for energy production, and lipid synthesis, which is crucial for the formation of membrane lipids, signaling molecules, and hormones. During disc degeneration, disruptions in lipid metabolism impair cellular structure and function, accelerating the degenerative process. The increase in lipid peroxidation within disc tissues is strongly linked to the progression of IVDD. Lipid peroxidation destabilizes membranes, damages organelles, and induces apoptosis along with inflammatory responses (Goupille et al., 1998). Studies have found that levels of lipid peroxidation products, such as malondialdehyde (MDA) and 4-hydroxynonenal (4-HNE), are significantly elevated in the discs of IVDD patients, serving as markers of oxidative damage (Setton and Chen, 2004). In addition, lipid metabolism reprogramming in IVDD involves pathways such as fatty acid synthesis, oxidation, and cholesterol metabolism, which are modulated throughout the degeneration process and influence cell survival and function.

### 4.4 Amino acid metabolism and IVDD

#### 4.4.1 Characteristics of amino acid metabolic reprogramming

Metabolic changes of amino acids such as glutamine and serine play important roles in IVDD (Cheng et al., 2021). Amino acid metabolism not only provides energy and precursors for biosynthesis but also affects cell survival and function by regulating intracellular redox status and signaling pathways (Paulusma et al., 2022). Studies have found significant changes in the levels of glutamine and serine in the disc cells of IVDD patients, suggesting metabolic reprogramming of these amino acid pathways (Shi et al., 2023).

#### 4.4.2 Metabolic changes of key amino acids

Glutamine plays a critical role in cellular energy metabolism and antioxidation. It is not only an anaplerotic substrate for the TCA cycle but also maintains cellular redox balance by generating glutathione (GSH) (DeBerardinis and Cheng, 2010; Yoo et al., 2020). In IVDD, alterations in glutamine metabolic pathways may affect antioxidant capacity and energy supply (Dong et al., 2024). Similarly, serine is crucial for nucleotide and protein synthesis, and its metabolic changes may impact cell growth and repair capacity (Wu et al., 2021).

### 4.5 Oxidative stress and mitochondrial function

#### 4.5.1 Mitochondrial dysfunction and its role in metabolic reprogramming

Mitochondria are the powerhouses of the cell, and their dysfunction plays a key role in IVDD. Studies have shown that mitochondrial membrane potential is decreased, and ATP production is reduced in the disc cells of IVDD patients, indicating severe mitochondrial impairment (Gruber et al., 2015; Song et al., 2023). Mitochondrial dysfunction affects energy metabolism and induces oxidative stress by releasing ROS (Zorov et al., 2014).

#### 4.5.2 Metabolic regulation of oxidative stress

Oxidative stress influences metabolic reprogramming and cell survival through various pathways. Excessive generation of ROS can lead to DNA damage, protein oxidation, and lipid peroxidation, triggering apoptosis and autophagy dysfunction (Kaarniranta et al., 2019). Additionally, oxidative stress regulates metabolic reprogramming of glycolysis, the TCA cycle, and lipid metabolism by modulating signaling pathways such as HIF-1α and NF-κB (Liu et al., 2018). Studies have found that the use of antioxidants can partially alleviate IVDD symptoms, indicating that oxidative stress is an important therapeutic target (Shao et al., 2021).

**FIGURE 2:**
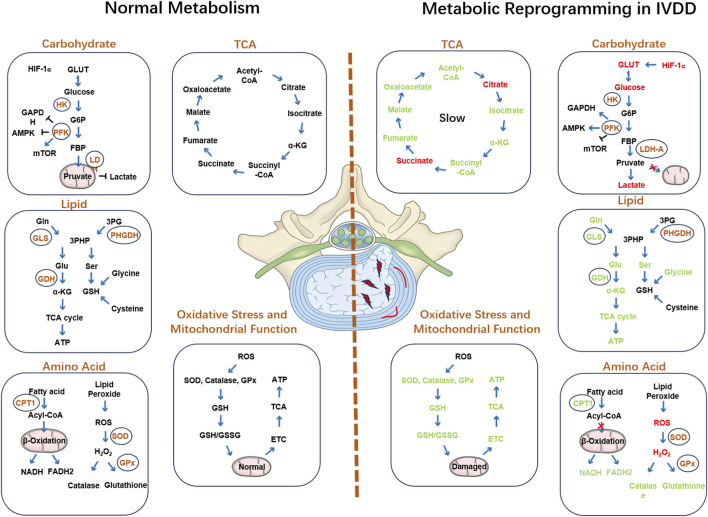
Comparison of metabolism in normal intervertebral discs and metabolic reprogramming in degenerated discs. The left panel illustrates the metabolic features of healthy discs, including balanced glycolysis, lipid metabolism, amino acid homeostasis, and preserved mitochondrial function. The right panel depicts metabolic reprogramming in degenerated discs, characterized by enhanced glycolysis, mitochondrial dysfunction, lipid peroxidation, altered amino acid metabolism, oxidative stress, inflammation, and ECM breakdown.

By systematically exploring the specific mechanisms of metabolic reprogramming in IVDD, we can gain a deeper understanding of the changes in metabolic pathways and their impact on intervertebral disc cells (Figure 2). These findings provide new perspectives for future research and facilitate the development of developing new therapeutic strategies to improve the treatment of IVDD (Table 1).

**TABLE 1:** Specific mechanisms of metabolic reprogramming in IVDD.

Metabolic pathway	Key changes	Key enzymes or molecules	References
Glycolysis and lactate metabolism	Increased glycolysis and lactate production. Hypoxia upregulates HIF-1α and GLUTs, enhancing glycolysis. Lactate lowers pH, exacerbating inflammation and matrix degradation	HIF-1α, GLUT, LDH, PFK, PK, MCT, inflammatory cytokines (IL-1β, TNF-α), HK2, PDK, Aldolase	Risbud and Shapiro (2014), Wang et al. (2020), Majmundar et al. (2010), Papandreou et al. (2006), Smith et al. (2011), Richardson et al. (2008)
TCA cycle alterations	Reduced TCA cycle activity, diminished ATP production, and mitochondrial dysfunction. Accumulation of intermediates like fumarate and malate	SDH, FH, IDH, citrate, succinate, ATP synthase, Aconitase, Malate dehydrogenase	Tendulkar et al. (2019), Lynch and Adams (2014), Im and Osborne (2011), Vander Heiden and DeBerardinis (2017), Zieba et al. (2016)
Lipid metabolism reprogramming	Increased lipid peroxidation and reactive lipid species accumulation. Altered phospholipid composition and cholesterol metabolism. Triggering apoptosis and inflammation	Lipid peroxides, arachidonic acid derivatives, MDA, 4-HNE, LOX, COX, PPARs, FAS, ACC, SREBPs	Setton and Chen (2004), Chen et al. (2022), Chen et al. (2023b)
Amino acid metabolism	Altered glutamine and serine metabolism, impacting energy balance and redox status. Disrupted BCAA metabolism affects mTOR signaling and protein synthesis	Glutamine, serine, GSH, GLS, PHGDH, PSAT1, SHMT, BCAT, GDH, ALT	Hanahan and Weinberg (2011), Stockwell et al. (2017), Sivanand and Vander Heiden (2020), Wu et al. (2021b), Zhang et al. (2024), Wu et al. (2024)
Oxidative stress and mitochondrial function	Mitochondrial dysfunction, increased ROS production, leading to cell apoptosis. Loss of mitochondrial membrane potential and reduced oxidative phosphorylation	ROS, mitochondrial membrane potential, SOD, GPx, MitoQ, CoQ10, NADPH oxidase, Catalase, Prxs	Zorov et al. (2014), Lin et al. (2021b), Wang et al. (2023b), Cao et al. (2022), Wang et al. (2022)

HIF-1α, Hypoxia-Inducible Factor 1-alpha; GLUT, glucose transporter; LDH, lactate dehydrogenase; PFK, phosphofructokinase; PK, pyruvate kinase; MCT, monocarboxylate transporter; IL-1β, Interleukin 1-beta; TNF-α, Tumor Necrosis Factor-alpha; HK2, Hexokinase 2; PDK, pyruvate dehydrogenase kinase; SDH, succinate dehydrogenase; FH, fumarate hydratase; IDH, isocitrate dehydrogenase; ATP, adenosine triphosphate; MDA, malondialdehyde; 4-HNE, 4-Hydroxynonenal; LOX, lipoxygenase; COX, cyclooxygenase; PPARs, Peroxisome Proliferator-Activated Receptors; BCAA, Branched-Chain Amino Acids; GSH, glutathione; GLS, glutaminase; PHGDH, phosphoglycerate dehydrogenase; PSAT1, Phosphoserine Aminotransferase 1; SHMT, serine hydroxymethyltransferase; BCAT, Branched-Chain Aminotransferase; GDH, glutamate dehydrogenase; ALT, alanine aminotransferase; SOD, superoxide dismutase; GPx, Glutathione Peroxidase; NADPH, nicotinamide adenine dinucleotide phosphate; Prxs, Peroxiredoxins.

## 5 Regulatory factors of metabolic reprogramming

### 5.1 Genetic factors

#### 5.1.1 Gene mutations and their impact on metabolic pathways

Genetic factors play a critical role in metabolic reprogramming and the development of IVDD. Specific gene mutations can lead to abnormal metabolic pathways, thereby promoting the progression of IVDD. For example, mutations in the COL9A2 and COL9A3 genes are closely associated with IVDD, as these mutations affect the metabolic state of intervertebral disc cells, leading to ECM degradation and loss of cell function (Kamali et al., 2021). Additionally, mutations in the MMPs and ADAMTS family genes have also been linked to the occurrence and development of IVDD, resulting in abnormal expression and activity of metabolic enzymes, further accelerating ECM degradation and cell damage (Roberts et al., 2006).

### 5.2 Environmental factors

#### 5.2.1 Nutritional status

Nutritional status has a significant impact on metabolic reprogramming. Malnutrition or nutritional imbalance can accelerate the progression of IVDD by affecting metabolic pathways. Studies have shown that intervertebral disc cells exhibit metabolic dysfunction, reduced cell viability, and ECM degradation when deprived of essential nutrients such as glucose, amino acids, and fatty acids (Yin et al., 2020). Nutritional supplements such as omega-3 fatty acids, vitamin D, and chondroitin sulfate have shown potential in improving disc health in some studies, indicating that regulating nutritional status may be an important means of preventing and treating IVDD (Huang et al., 2019).

#### 5.2.2 Physiological stress

Physiological stress, such as mechanical stress and oxidative stress, significantly impacts metabolic reprogramming. Excessive mechanical stress on the intervertebral disc leads to ECM degradation and cell apoptosis, further contributing to the progression of IVDD (Fu et al., 2021). Additionally, oxidative stress disrupts normal metabolic functions by producing excessive ROS, causing mitochondrial damage and reduced ATP production (Feng et al., 2017). Studies have found that antioxidants such as vitamins C and E can improve the metabolic state and function of intervertebral disc cells by alleviating oxidative stress (Smith, 2010).

### 5.3 Signaling pathways and transcription factors

#### 5.3.1 Key signaling pathways

The mTOR and AMPK pathways play critical regulatory roles in metabolic reprogramming and IVDD. mTOR is a key metabolic regulator that promotes protein synthesis, inhibits autophagy, and thereby regulates cell growth and metabolism (Chen H. W. et al., 2023). In IVDD, overactivation of the mTOR pathway leads to ECM degradation and cell apoptosis. AMPK is an energy-sensing kinase that maintains cellular energy balance by activating autophagy and regulating lipid metabolism (Wang et al., 2021). Studies have shown that activation of AMPK can slow the progression of IVDD by regulating metabolic reprogramming (Lin et al., 2021).

#### 5.3.2 Transcription factors

Transcription factors such as c-Myc and HIF-1α influence the development of IVDD by regulating the expression of metabolism-related genes. c-Myc is an important transcription factor that promotes rapid cell growth and division by regulating glycolysis and amino acid metabolism (Dang, 2012). In IVDD, abnormal expression of c-Myc leads to metabolic dysregulation and ECM degradation (Zhou et al., 2021). HIF-1α is activated in a hypoxic environment and enhances cellular dependence on glycolysis by upregulating the expression of glycolysis-related genes (Majmundar et al., 2010). Studies have shown that the activation of HIF-1α plays a protective role in IVDD by promoting metabolic reprogramming and helping cells adapt to a hypoxic environment (Li et al., 2021).

By conducting in-depth research on the regulatory factors of metabolic reprogramming, we can gain a better understanding of the pathogenesis of IVDD and identify potential therapeutic targets. These findings not only provide new perspectives for future research but also facilitate the development of developing new treatment strategies (Table 2).

**TABLE 2:** Regulatory factors of metabolic reprogramming in IVDD.

Metabolic pathway	Key changes	Key enzymes or molecules	References
Genetic factors	Mutations in COL9A2, COL9A3 lead to ECM degradation and cell death. Altered MMPs and ADAMTS expression increases ECM catabolism and inflammation. VDR and IL-1 polymorphisms influence IVDD susceptibility	COL9A2, COL9A3, MMPs, ADAMTS enzymes, VDR, IL-1	Xie et al. (2021), Zhao et al. (2015), Mashayekhi et al. (2018), Solovieva et al. (2006), Richardson et al. (2008), Solovieva et al. (2004)
Environmental factors	Nutrient deficiency (glucose, amino acids, lipids) impacts cell function. Mechanical stress causes ECM damage and apoptosis. Smoking and pollutants exacerbate oxidative stress and inflammation	Glucose, amino acids, lipids, mechanical stress, smoking, pollutants	Ashinsky et al. (2020), Lener et al. (2020), Ariga et al. (2003), Shi et al. (2022), Jing et al. (2021)
Signaling pathways	mTOR promotes anabolism, inhibits autophagy. AMPK promotes catabolism, autophagy under stress. Wnt/β-catenin affects cell proliferation and differentiation	mTOR, AMPK, anabolism, catabolism, autophagy, Wnt/β-catenin	Chen et al. (2023a), Yang et al. (2019), Zhang et al. (2021), Lu et al. (2023)
Transcription factors	HIF-1α regulates glycolysis in hypoxia. c-Myc drives glutamine metabolism and nucleotide synthesis. NF-κB mediates inflammation and metabolic reprogramming. SOX9 influences ECM synthesis and differentiation	HIF-1α, c-Myc, NF-κB, SOX9	Bae et al. (2024), Kim et al. (2021), Liu et al. (2012), Londhe et al. (2018), Tsingas et al. (2020)
Metabolic enzymes	Increased glycolytic enzyme activity (HK2, PFK, LDH) promotes glycolysis. Altered TCA cycle enzymes (SDH, FH) affect ATP production. Upregulated lipid metabolism enzymes (FAS, ACC) lead to lipid accumulation. Dysregulated autophagy enzymes (Beclin-1, LC3-II)	HK2, PFK, LDH, SDH, FH, FAS, ACC, Beclin-1, LC3-II	Fantin et al. (2006), Wu et al., 2021a; Hardie (2011), Cao et al. (2022), Feng et al. (2025)

COL9A2, Collagen Type IX, Alpha 2; COL9A3, Collagen Type IX, Alpha 3; MMPs, Matrix Metalloproteinases; ADAMTS, A Disintegrin and Metalloproteinase with Thrombospondin Motifs; VDR, Vitamin D Receptor; IL-1, Interleukin 1; ECM, extracellular matrix; AMPK, AMP-activated Protein Kinase; mTOR, mechanistic target of rapamycin; Wnt/β-catenin, Wingless-related integration site/β-catenin signaling pathway; HIF-1α, Hypoxia-Inducible Factor 1-alpha; c-Myc, Cellular Myc Oncogene; NF-κB, Nuclear Factor kappa-light-chain-enhancer of activated B cells; SOX9, SRY-Box Transcription Factor 9; LC3-II, Microtubule-Associated Proteins 1A/1B Light Chain 3B.

## 6 Potential therapeutic targets of metabolic reprogramming

### 6.1 Metabolic enzyme inhibitors

#### 6.1.1 Glycolytic enzyme inhibitors

In the pathological process of IVDD, abnormal activation of key enzymes in the glycolytic pathway, such as HK and LDH, is a significant factor leading to metabolic reprogramming. Inhibiting these key enzymes can effectively slow or reverse the progression of IVDD. Research by Mathupala et al. demonstrated that inhibitors of HK2 can reduce lactate production, thereby decreasing cellular acidification and damage (Mathupala et al., 2009). Additionally, studies by Fantin et al. showed that LDH inhibitors significantly slowed ECM degradation in experimental models (Fantin et al., 2006). Further studies have explored the potential of targeting other glycolytic enzymes, such as phosphofructokinase (PFK) and pyruvate kinase M2 (PKM2). Inhibiting PFK has been shown to reduce the glycolytic flux, thereby lowering lactate levels and minimizing the acidic microenvironment that exacerbates disc cell degeneration. Similarly, targeting PKM2 not only reduces glycolytic activity but also affects other pathways like the pentose phosphate pathway (PPP), which is involved in nucleotide synthesis and redox balance. By modulating these pathways, PKM2 inhibitors can potentially reduce oxidative stress and promote cell survival in degenerated discs (Fantin et al., 2006).

#### 6.1.2 TCA cycle enzyme inhibitors

Inhibitors of key enzymes in the TCA cycle, such as SDH and FH, also show potential in inhibiting ECM degradation and reducing apoptosis in intervertebral disc cells. Research by King et al. found that SDH and FH inhibitors can effectively reduce metabolic disorders and oxidative stress in IVDD (Ying et al., 2012). Recent research has also highlighted the therapeutic potential of inhibiting isocitrate dehydrogenase (IDH), which can lead to the accumulation of 2-hydroxyglutarate (2-HG), a metabolite that can interfere with cellular redox balance and epigenetic regulation. Targeting IDH, particularly in cases where IDH mutations are present, can help restore normal metabolic function and reduce the aberrant epigenetic changes associated with disc degeneration (King et al., 2006). Furthermore, targeting the alpha-ketoglutarate dehydrogenase complex has been proposed as a strategy to modulate the production of ROS and mitigate oxidative damage, thereby preserving mitochondrial function and promoting disc cell viability (Ward et al., 2010).

### 6.2 Metabolic regulators

#### 6.2.1 AMPK activators

AMPK activators such as AICAR and metformin help maintain cellular energy balance and reduce damage to intervertebral disc cells by activating autophagy and lipid metabolism. Zhou et al. demonstrated that AMPK activators can enhance the survival rate of intervertebral disc cells and slow ECM degradation (Zhou et al., 2001). Further research by Jiang et al. showed that AMPK activators significantly improved IVDD symptoms in animal models (Xiang et al., 2019).

#### 6.2.2 mTOR inhibitors

mTOR inhibitors, such as rapamycin, reduce metabolic load and alleviate symptoms of disc degeneration by inhibiting protein synthesis and cell growth. Sun et al. found that rapamycin significantly reduced inflammation and oxidative stress in IVDD (Wullschleger et al., 2006). Research by Laplante and Sabatini showed that mTOR inhibitors also protect intervertebral disc cells by inhibiting inflammatory responses and oxidative stress.

### 6.3 Antioxidants and mitochondrial protectors

#### 6.3.1 Antioxidants

Antioxidants such as vitamins C and E and N-acetylcysteine (NAC) mitigate oxidative stress-induced damage to intervertebral disc cells by neutralizing excessive ROS. Liu et al. **have demonstrated** that NAC significantly **reduces** oxidative stress and inflammation in IVDD experimental models (Riegger and Brenner, 2019). In addition to these well-known antioxidants, emerging research has identified other compounds with potent antioxidant properties, such as resveratrol and curcumin. Resveratrol, a polyphenolic compound found in red wine, has been shown to activate the SIRT1 pathway, leading to enhanced antioxidative defenses and reduced inflammation in disc cells. Similarly, curcumin, derived from turmeric, has been found to inhibit the NF-κB signaling pathway, thereby reducing the expression of pro-inflammatory cytokines and ROS production. These findings suggest that these antioxidants may help attenuate the degenerative processes in IVDD by protecting against oxidative damage and modulating inflammatory responses.

#### 6.3.2 Mitochondrial protectors

Mitochondrial protectors such as MitoQ and CoQ10 maintain mitochondrial membrane potential and ATP production, thereby preserving mitochondrial function and improving the energy metabolism of intervertebral disc cells. Research by Smith et al. found that MitoQ significantly improved mitochondrial function and cell survival rates in animal models of IVDD (Murphy and Smith, 2007). Studies by Cao et al. showed that CoQ10 effectively protects intervertebral disc cells from oxidative stress-induced damage (Cao et al., 2022). Furthermore, other mitochondrial-targeted antioxidants, such as SkQ1 and SS-31, have shown promising results in preserving mitochondrial integrity and reducing apoptosis in disc cells. SkQ1, a plastoquinone derivative, specifically targets mitochondria and has been demonstrated to reduce mitochondrial ROS production and inhibit the opening of the mitochondrial permeability transition pore (mPTP). SS-31, a mitochondrial-targeting peptide, has been shown to enhance mitochondrial bioenergetics and reduce oxidative stress. These agents not only protect against mitochondrial dysfunction but also have the potential to ameliorate the overall metabolic dysregulation seen in IVDD (Liu et al., 2022).

By delving into the potential therapeutic targets of metabolic reprogramming, we can identify more effective strategies for treating IVDD. These metabolic enzyme inhibitors, metabolic regulators, and antioxidants not only slow the progression of disc degeneration but also enhance the survival and function of intervertebral disc cells by improving their metabolic state (Table 3).

**TABLE 3:** **Metabolic reprogramming as a potential therapeutic target in IVDD**.

Therapeutic target	Mechanism of action	Potential benefits	Key molecules or drugs	References
Glycolytic inhibitors	Inhibit key glycolytic enzymes to reduce lactate production and acidification. Target HIF-1α to decrease glycolysis under hypoxia	Decrease inflammation and ECM degradation. Reduce cellular acidification and apoptosis	2-Deoxy-D-glucose (2-DG), FX11, HIF-1α inhibitors	Wu et al. (2021a), Richardson et al. (2008), Majmundar et al. (2010), Fantin et al. (2006), Shi et al. (2022), Ashinsky et al. (2020)
TCA Cycle modulators	Enhance TCA cycle activity and ATP production. Use modulators to restore normal function of SDH and FH	Improve cellular energy status and reduce metabolic stress. Enhance cell survival and function	Dichloroacetate (DCA), SDH activators, FH stabilizers	James et al. (2017), Matsuhashi et al. (2015), Gibala and Saltin (1999), Tendulkar et al. (2019), Wu et al. (2025), Schaffer et al. (2011)
Lipid metabolism regulators	Regulate lipid metabolism to reduce lipid peroxidation and accumulation. Target enzymes like FAS and ACC	Decrease lipid peroxidation and cellular damage. Improve membrane integrity and reduce inflammation	Orlistat, FAS inhibitors, ACC inhibitors	Liu and Sabatini (2020), Lamming and Sabatini (2013), Li et al. (2022), Fan et al. (2023), Jones and Infante (2015)
Amino acid metabolism modulators	Modulate glutamine and serine metabolism to restore energy balance and reduce oxidative stress. Inhibit enzymes like GLS and PHGDH	Restore redox balance and reduce oxidative stress. Enhance cellular energy and reduce apoptosis	Glutaminase, PHGDH	Wu et al. (2024), Wu et al. (2021b), Jackson et al. (2025), Stockwell et al. (2017), Sivanand and Vander Heiden (2020)
Antioxidants and mitochondrial protectors	Scavenge reactive oxygen species (ROS) and protect mitochondrial function. Use antioxidants like MitoQ and CoQ10	Protect cells from oxidative damage and apoptosis. Maintain mitochondrial function and energy production	MitoQ, CoQ10, N-acetylcysteine (NAC)	Kang et al. (2020), Sun et al. (2023), González et al. (2009), Murphy, 2009; Noh et al. (2013), Shi et al. (2024)

HIF-1α, Hypoxia-Inducible Factor 1-alpha; 2-DG, 2-Deoxy-D-glucose; TCA, tricarboxylic acid cycle; SDH, succinate dehydrogenase; FH, fumarate hydratase; FAS, fatty acid synthase; ACC, Acetyl-CoA carboxylase; PHGDH, phosphoglycerate dehydrogenase; GLS, glutaminase; ROS, reactive oxygen species; NAC, N-Acetylcysteine; MitoQ, Mitoquinone (a mitochondria-targeted antioxidant); CoQ10, Coenzyme Q10.

## 7 Summary and outlook

### 7.1 Summary of the current research status

In recent years, substantial progress has been made in understanding the role of metabolic reprogramming in IVDD. Current evidence suggests that metabolic reprogramming is closely involved in IVDD progression and is associated with multiple metabolic pathways and regulatory factors. This review summarizes the major pathological mechanisms of IVDD, including apoptosis, autophagy, inflammatory responses, ECM degradation, and oxidative stress. It also discusses alterations in glucose, lipid, and amino acid metabolism, as well as the genetic and environmental factors, signaling pathways, and transcriptional regulators involved in metabolic reprogramming. Overall, this review provides an integrated overview of the metabolic mechanisms implicated in IVDD and highlights potential therapeutic targets, including metabolic enzyme modulation, metabolic regulators, and antioxidants.

### 7.2 Future research directions

Despite recent progress, several important questions regarding metabolic reprogramming in IVDD remain unresolved. Future research should focus on the following directions.

#### 7.2.1 In-depth study of specific mechanisms

Further studies are needed to clarify the specific mechanisms of metabolic reprogramming and its precise role in IVDD. For example, how specific metabolic pathways can be modulated to delay or reverse IVDD progression requires further investigation (Yan et al., 2021). In addition, the interactions among different metabolic pathways and their combined effects warrant further study. A better understanding of the crosstalk between anabolic and catabolic processes in disc cells may help identify potential therapeutic targets. It will also be important to investigate the role of mitochondrial dynamics, including fission and fusion, in cellular energy homeostasis and their relationship with apoptosis and autophagy. Moreover, the epigenetic regulation of metabolic enzymes, such as histone modifications and DNA methylation, may provide further insight into the long-term metabolic alterations associated with disc degeneration.

#### 7.2.2 Clinical translation

Research on metabolic reprogramming may provide potential therapeutic strategies and drug targets for IVDD. Future studies should focus on translating basic research findings into clinical applications. For example, high-throughput screening may facilitate the identification of novel metabolic regulators, and agents targeting specific metabolic pathways may help restore metabolic homeostasis in intervertebral disc cells (Zhang et al., 2024). In addition, the development of non-invasive biomarkers for early detection of metabolic alterations in IVDD may improve the timing of intervention. Clinical studies evaluating the efficacy and safety of metabolic modulators, such as glycolytic inhibitors or mitochondrial protectors, will also be important. Personalized approaches based on metabolic characteristics and genetic predisposition may further improve treatment precision. At the same time, the potential adverse effects and off-target consequences of metabolic intervention should be carefully evaluated to ensure safety (Song et al., 2024).

#### 7.2.3 Comprehensive treatment strategies

Given the multifactorial nature of IVDD, future therapeutic strategies may require the integration of multiple approaches. For example, combining metabolic regulation with anti-inflammatory treatment and mechanical stress modulation may produce better therapeutic effects. The combination of physical therapies with pharmacological interventions targeting metabolic and inflammatory pathways may provide a more comprehensive treatment strategy. In addition, dietary interventions and nutritional support may also contribute to metabolic homeostasis and inflammation control in disc cells. Furthermore, combining regenerative approaches, such as stem cell therapy or tissue engineering, with metabolic intervention may improve disc repair and functional recovery.

#### 7.2.4 Application of new technologies

Emerging technologies in molecular biology and gene editing, such as CRISPR-Cas9, may be useful for investigating gene regulation and metabolic reprogramming in IVDD. These approaches may allow selective manipulation of genes involved in key metabolic pathways, thereby facilitating mechanistic studies. In addition, organoid models and advanced three-dimensional bioprinting may provide more representative *in vitro* systems for studying disc metabolism and evaluating therapeutic interventions. Single-cell sequencing technologies may further improve understanding of disc cell heterogeneity and metabolic states, thereby supporting the development of more precise therapeutic strategies. As these technologies continue to evolve, ethical and regulatory issues should also be carefully considered to support their safe and appropriate clinical translation.

In summary, metabolic reprogramming is an important component of IVDD research and may provide a useful basis for further mechanistic and translational studies. Future work should continue to clarify its role in disc degeneration and evaluate its potential relevance for clinical intervention.
